# Clark's nutcracker forest community visitation: Whitebark pine maintains a keystone seed disperser

**DOI:** 10.1002/ece3.10813

**Published:** 2023-12-21

**Authors:** Thomas H. McLaren, Diana F. Tomback, Nels Grevstad, Michael B. Wunder, Walter Wehtje, Lauren E. Walker, Douglas W. Smith

**Affiliations:** ^1^ Department of Integrative Biology University of Colorado Denver Denver Colorado USA; ^2^ Department of Mathematics and Statistics Metropolitan State University of Denver Denver Colorado USA; ^3^ Ricketts Conservation Foundation Bondurant Wyoming USA; ^4^ Yellowstone National Park Yellowstone Center for Resources Yellowstone National Park Wyoming USA; ^5^ Present address: Klamath Bird Observatory Ashland Oregon USA; ^6^ Present address: United States Geological Survey Eastern Ecological Science Center Laurel Maryland USA

**Keywords:** Clark's nutcracker, cone production, forest communities, hierarchical distance sampling model, seed caching whitebark pine, white pine blister rust, Yellowstone National Park

## Abstract

Clark's nutcrackers (*Nucifraga columbiana*) are obligate seed dispersers for whitebark pine (*Pinus albicaulis*), but they frequently use other conifer seed resources because of annual variability in cone production or geographic variation in whitebark pine availability. Whitebark pine is declining from several threats including white pine blister rust, leading to potential population declines in the nutcracker and the pine. We hypothesize that where there are few additional seed resources, whitebark pine becomes the key and limiting resource supporting nutcracker populations. We investigated how nutcrackers use coniferous forest community types within Yellowstone National Park to determine potential seed resources and the importance of whitebark pine. We established sites representing five forest community types, including whitebark pine, lodgepole pine (*P. contorta*), Engelmann spruce (*Picea engelmannii*), limber pine (*P. flexilis*), and Douglas‐fir (*Pseudotsuga menziesii*). Each transect annually generated nutcracker point counts, conifer cone production indices, community composition data, and seed resource use observations. We compared hierarchical distance sampling models, estimating nutcracker density and its relationship to forest community type, seed harvesting time‐period, year, study site, and cone seed energy. We found cone production varied across years indicating annual variability in energy availability. Nutcracker density was best predicted by forest community type and survey time‐period and was highest in whitebark pine stands during the mid‐harvesting season. Nutcracker density was comparatively low for all other forest community types. This finding underscores the importance of whitebark pine as a key seed resource for Clark's nutcracker in Yellowstone National Park. The decline of whitebark pine potentially leads to a downward spiral in nutcrackers and whitebark pine, arguing for continued monitoring of nutcrackers and implementation of restoration treatments for whitebark pine.

## Graphical abstract

The coevolved mutualism between whitebark pine (*Pinus albicaulis*) and Clark's nutcracker (*Nucifraga columbiana*) is well documented, but our understanding of nutcracker use of additional conifer seed resources is lacking. Data collected over 3 years in Yellowstone National Park suggest that whitebark pine was the only seed resource that nutcrackers visited regularly and that bird density was highest during mid‐September when whitebark pine seeds ripen. In areas without additional seed resources to sustain populations of nutcrackers, we hypothesize that Clark's nutcracker populations may be more directly linked to whitebark pine seed production.

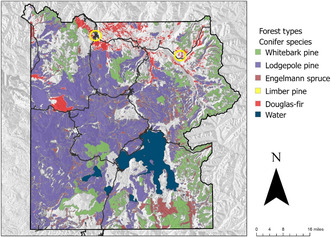

## 1 INTRODUCTION

**FIGURE 1:**
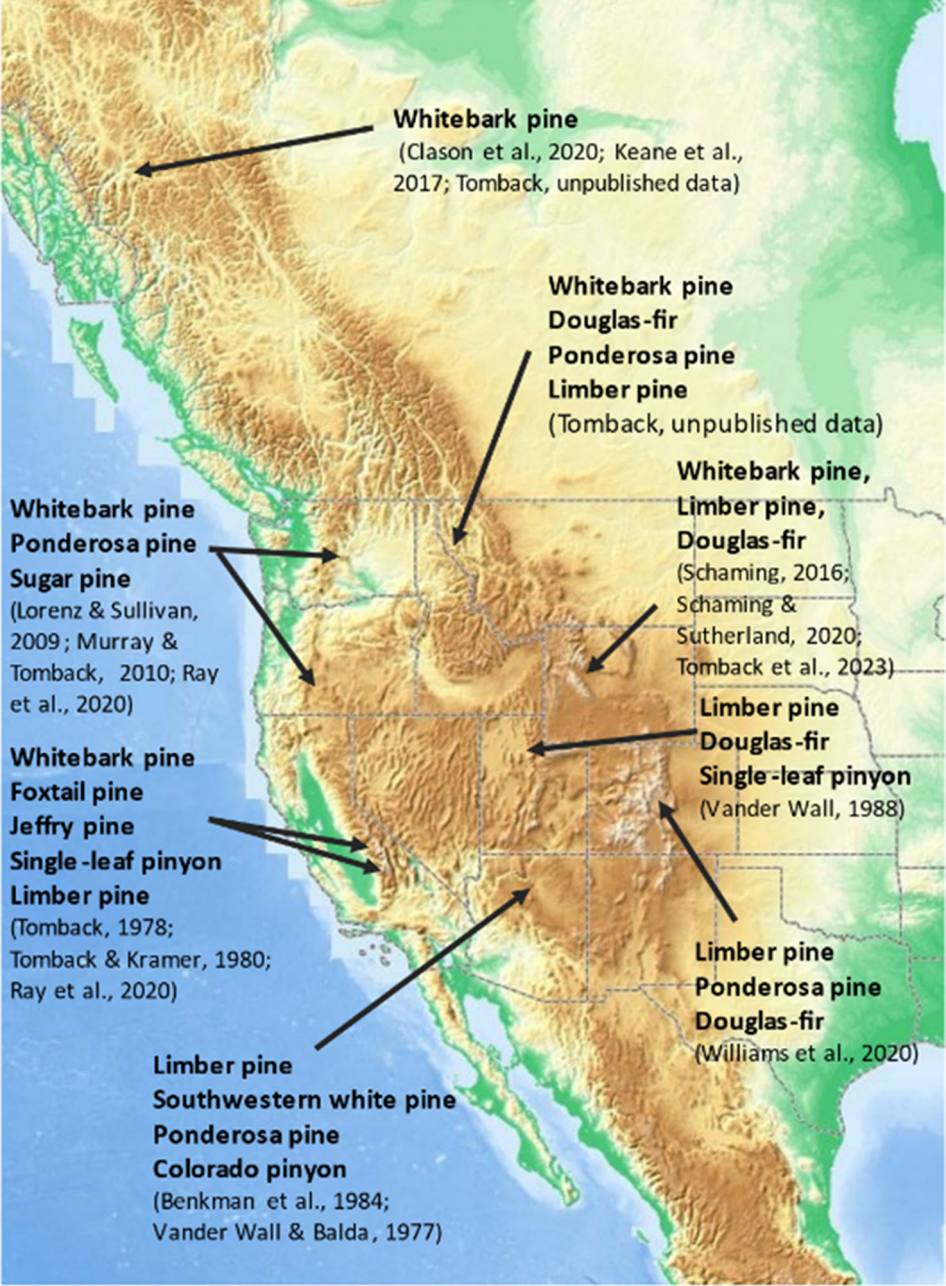
Seed resources used by Clark's nutcracker (*Nucifraga columbiana*) across its range, according to the cited studies for each region. The reliable range of the nutcracker ends south of the Sacramento Mountains and Mogollon Plateau, New Mexico, and at its northern edge at about 55° (Tomback, 1998).

Clark's nutcracker (*Nucifraga columbiana*) inhabits the higher mountains of western North America, ranging from northern Baja California north through Canada (Tomback, 1998). Each fall, Clark's nutcrackers each cache tens of thousands of conifer seeds from different conifer species for consumption during resource‐scarce months of late‐winter and spring (Hutchins & Lanner, 1982; Lorenz et al., 2008; Samano & Tomback, 2003; Tomback, 1978, 1982; Vander Wall & Balda, 1977; Williams et al., 2020). During years of high cone production, they cache more seeds than they consume, and some birds may not survive or remain locally to use their caches. Seeds in unretrieved caches have the potential to germinate and contribute to conifer forest regeneration (Tomback, 1982; Tomback et al., 2001). Because nutcrackers harvest and cache the seeds of many western conifers, they contribute to their regeneration and thus serve the role of keystone seed dispersers, and “mobile links” connecting spatially separated conifer populations across the United States and Canada (Tomback, 1998; Tomback & Kendall, 2001; Williams et al., 2020) (Figure 1). This seed caching behavior provides an important ecosystem service that benefits conifers by transporting their seeds over distances much greater than can be achieved by wind‐dispersal (e.g., Greene & Johnson, 1996; Lorenz & Sullivan, 2009; McCaughey et al., 1986; Schaming, 2016; Tomback et al., 1990). Recent radio‐tracking and GPS data indicate that Clark's nutcrackers are highly mobile (Lorenz & Sullivan, 2009; Schaming, 2016; Tomback et al., unpublished); and nutcracker population genetic data indicates panmixia, likely resulting from long‐distance individual movements (Dohms & Burg, 2013).

Whitebark pine (*Pinus albicaulis*), has evolved an obligate dependency on Clark's nutcracker for seed dispersal, a product of coevolution and coadaptation (Lanner, 1982, 1990; Tomback, 1983; Tomback & Linhart, 1990). The whitebark pine traits that facilitate this interaction include cones that develop in horizontally oriented whorls on vertically directed branches and remain closed after maturity, and large, wingless seeds that preclude dispersal by wind (Lanner, 1982; Tomback & Linhart, 1990). Nutcracker adaptations include a long, sharp bill to extract seeds from cones, a sublingual throat pouch to transport seeds, and a highly accurate spatial memory to aid cache retrieval (Tomback, 1978, 1980; Vander Wall, 1982).

Whitebark pine is an ecological keystone and foundation forest species that inhabits subalpine and treeline elevations in the western United States and Canada (Arno & Hoff, 1990; Degrassi et al., 2019; Ellison et al., 2005; Tomback & Achuff, 2010). Whitebark pine is rapidly declining across much of its range in both the United States and Canada (Goeking & Izlar, 2018; Tomback & Achuff, 2010; Tomback et al., 2001), prompting the recent listing of whitebark pine as threatened under the Endangered Species Act by the U.S. Fish and Wildlife Service (2022). White pine blister rust (WPBR), a disease caused by the exotic pathogen *Cronartium ribicola*, is the primary threat to whitebark pine; this pathogen kills trees by girdling stems and branches, which prevents the transport of water and nutrients (McDonald & Hoff, 2001; Tomback & Achuff, 2010; U.S. Fish and Wildlife Service, 2022). Other threats to whitebark include recent, regional outbreaks of mountain pine beetles (*Dendroctonus ponderosae*) and altered fire regimes both from historical fire exclusion and from more frequent and severe fires (Gibson et al., 2008; Higuera et al., 2021; Tomback & Achuff, 2010). Climate warming is also changing the distribution of whitebark pine at local and regional scales and drives native bark beetle outbreaks, alters fire regimes, and potentially enables *C. ribicola* to invade more extreme environments (Chang et al., 2014; Dudney et al., 2021; Logan & Powell, 2004; Thoma et al., 2019; Tomback et al., 2016). Recent analysis of Forest Inventory and Analysis data (USDA Forest Service) determined more than 50% overall of standing whitebark pine to be dead, with the Northern United States Rocky Mountain populations in greatest decline (Goeking & Izlar, 2018; Goeking & Windmuller‐Campione, 2021).

Most conifer species, including whitebark pine, are known for high interannual variation in cone production (Crone et al., 2011; Table 3 in Krugman & Jenkinson, 1974). For nutcrackers, this variability necessitates having more than one conifer species within a geographic region as seed resources (e.g., Lorenz et al., 2011; Tomback, 1978; Vander Wall, 1988; Williams et al., 2020) (Figure 1). Across their range, Clark's nutcrackers prefer the most energetically rewarding seed sources—usually conifers with large, wingless seeds, including whitebark pine (Tomback, 1978), limber pine (Benkman et al., 1984; Tomback & Kramer, 1980; Vander Wall, 1988), southwestern white pine (Benkman et al., 1984; Samano & Tomback, 2003), Colorado pinyon (*Pinus edulis*) (Vander Wall & Balda, 1977), and single‐leaf pinyon (*Pinus monophylla*) (Tomback, 1978; Vander Wall, 1988). They also utilize as seed resources the other species listed in Figure 1, depending on local availability and annual cone production, and move from one seed resource to another following cone ripening phenology (e.g., Ray et al., 2020; Samano & Tomback, 2003; Tomback, 1978; Williams et al., 2020). During years of low cone production for the earlier ripening whitebark pine and limber pine, nutcrackers converge on later‐ripening resources such as ponderosa pine (*Pinus ponderosa*) and Douglas‐fir (*Pseudotsuga menziesii*) (e.g., Williams et al., 2020). This suggests that the availability of several conifer seed resources on the landscape may be important for maintaining regional Clark's nutcracker populations. In years when cone production is sparse or fails for all seed resources, nutcrackers may emigrate from a region (e.g., Davis & Williams, 1957, 1964; Fisher & Myres, 1980; Tomback, 1998; Vander Wall et al., 1981). Without additional seed resources, regional nutcracker carrying capacity may be based on the annual cone production of one or two conifer species (Tomback & Linhart, 1990); these nutcracker populations may be vulnerable to substantial decline if annual seed production is disrupted by insect infestation or disease.

The availability of multiple seed resources for use by Clark's nutcrackers varies geographically (Tomback, 1998) (Figure 1). For example, in the Southwestern United States, Clark's nutcrackers have available up to three pines with large, wingless seeds as well as ponderosa pine and Douglas‐fir (e.g., Benkman et al., 1984; Vander Wall & Balda, 1977). The Sierra Nevada may have the highest seed resource diversity: Across the east and west slopes, there are three pines with large, wingless seeds, three pines with large, winged seeds, and two pines and Douglas‐fir, with small, winged seeds (e.g., Ray et al., 2020; Tomback, 1978; Tomback & Kramer, 1980; D. F. Tomback, T. H. McLaren, W. Wehtje, L. E. Walker, D. W. Smith, unpublished data). Along the east slope of the northern Colorado Front Range, limber pine is the only broadly distributed seed resource with large, wingless seeds; but limber pine and Rocky Mountain bristlecone pine (*Pinus aristata*) are widely available in scattered stands, Colorado pinyon pine forms expansive stands at lower treeline on the west slope, and Douglas‐fir and ponderosa pine are widely available throughout the state (e.g., Tomback, 1998; Williams et al., 2020).

Given the nutcracker requirement for multiple seed resources within a region, the northern‐most limits to whitebark pine's range may result from lack of sufficient seed resources for a dependable population of Clark's nutcrackers (e.g., Clason et al., 2020; Keane et al., 2017) and lower cone production and seed viability (S. Haeussler, personal communication) rather than from climate. We predict nutcrackers will have higher dependence on whitebark pine annual cone production where additional seed resources are limited both in number and distribution. Similarly, we predict that whitebark pine seed production has relatively higher influence on annual nutcracker visitation and abundance where additional seed resources are limited.

**FIGURE 2:**
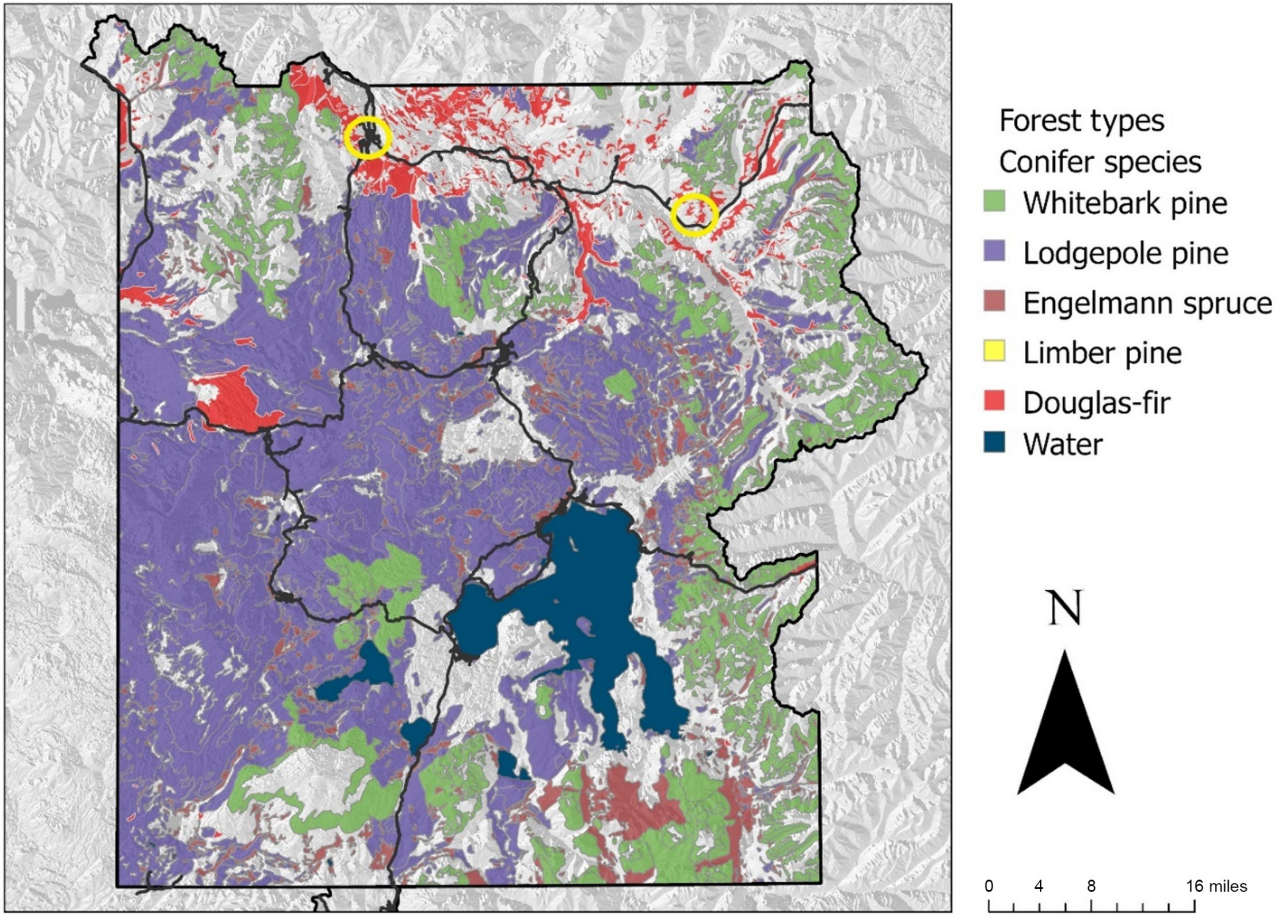
Forest types of Yellowstone National Park overlaid on a topographical map. Data accessed from the National Park Service IRMA database shows a simplified version of forest habitat types (1992). Areas on map designated as “Water” represent the major lakes within the park. We added locations of our two known limber pine stands on the forest types map layer, indicated by yellow circles; these forest communities are too spatially limited to be visible on the map at this scale. Limber pine also has a scattered distribution within the park along stream and river banks and on steep, rocky slopes (D. F. Tomback, T. H. McLaren, W. Wehtje, L. E. Walker, D. W. Smith, personal observations).

Whitebark pine is widely distributed in the Greater Yellowstone Ecosystem (GYE) (Figure 2) but declining in the region over the last 15 years. The recent mountain pine beetle outbreak killed up to 90% of cone‐bearing whitebark pine in some watersheds in the GYE (Logan et al., 2010; MacFarlane et al., 2013). Long‐term monitoring of whitebark pine health in the GYE indicates current overall WPBR infection rates between 14% and 26% (Shanahan et al., 2016, 2021). Blister rust was initially slow to invade the GYE, and infection rates to date are lower than in the Northern Rockies but ramping up (Goeking & Izlar, 2018; McDonald & Hoff, 2001). Yellowstone National Park resource managers (e.g., coauthors LEW, DWS) are concerned about maintaining populations of Clark's nutcracker and persistence of whitebark pine, and both advocated for and helped initiate this study. Trends in nutcracker abundance within the park have not been monitored, leading to uncertainty about the species' population status and resource use patterns.

In Yellowstone National Park, little is known about nutcracker use of conifer forest types other than whitebark pine (Tomback et al., 2023), and the only other seed resource known to be used by nutcrackers in the GYE is Douglas‐fir (Schaming, 2016). Nutcrackers have been observed caching Douglas‐fir seeds in Colorado (Williams et al., 2020) and also utilize dense Douglas‐fir forest communities for nesting habitat (Mewaldt, 1956; Schaming, 2016; Schaming & Sutherland, 2020; Tomback, 1998) and for shelter (Williams et al., 2020). Other conifer seed resources found in the park, such as limber pine, may potentially be used by nutcrackers but have not been examined. Determining whether nutcrackers utilize other seed resources in Yellowstone National Park and across the GYE potentially provides information about the magnitude of impact of whitebark pine loss on nutcracker populations, and the influence this may have on the nutcracker–whitebark mutualism (Tomback et al., 2023).

Recent studies show Clark's nutcrackers are strongly associated with whitebark and limber pine forest communities. Radio‐tracking individual birds in the Cascade Range, Washington, allowed Lorenz and Sullivan (2009) to link nutcracker space‐use with seed resource availability. In Rocky Mountain National Park, Colorado, Williams et al. (2020) found that nutcrackers routinely visited limber pine during seed harvest with little yearly variation in numbers. Ray et al. (2020) analyzed multiyear point count data from four national parks in the Sierra‐Cascade region, which suggested a decline in nutcracker abundance over time (Ray et al., 2017). They found that nutcracker density was positively related to whitebark pine cover in Sequoia‐Kings Canyon and Yosemite National Parks, and that trends in Mount Rainier National Park indicated declines in nutcracker density as WPBR incidence increased. These results suggest that nutcracker abundance is already in decline in some parts of the species' range and that increased WPBR prevalence may be a contributing factor.

In this study, we examine the importance of whitebark pine communities and four other forest community types in predicting Clark's nutcracker visitation in Yellowstone National Park. Yellowstone National Park is a region where only three of the known nutcracker seed resources occur, and two—Douglas‐fir and limber pine—are limited in extent. The larger context of our work is nutcracker carrying capacity in Yellowstone National Park and, by extrapolation, across the Greater Yellowstone region, and whether it may be more closely tied to whitebark pine than in regions with a greater diversity and abundance of known nutcracker seed resources. In other words, will a decline in whitebark pine in Yellowstone National Park lead to a decline in nutcracker populations and thus a spiraling decline in whitebark pine regeneration and disruption of the nutcracker–whitebark pine mutualism (e.g., Tomback & Kendall, 2001)? The broader application is to determine if Clark's nutcracker populations in regions with fewer seed resources are more dependent on whitebark pine and at greater risk from whitebark pine extirpation.

We investigated the following questions within the larger research context: (1) Which forest community types do Clark's nutcrackers frequent within Yellowstone National Park during late summer and fall seed ripening, seed harvest, and seed caching activities (late July–October)? Do some nutcrackers remain in the park during late fall and winter? (2) How do survey timing, cone ripeness, and magnitude of cone production influence estimates of nutcracker visitation and habitat use? and (3) In addition to whitebark pine, which seed resources do nutcrackers use within the park?

We addressed these questions with a hierarchical model‐based approach, using distance sampling to account for imperfect detectability, and we compared competing models using an information criterion approach (Burnham & Anderson, 2002; Kéry & Royle, 2015; Sillett et al., 2012). Annual variation in cone production is an important variable included in this study not addressed to date by most other Clark's nutcracker abundance studies (but see Williams et al., 2020). Most importantly, we believe that elucidating nutcracker forest habitat and seed resource use within Yellowstone National Park, and their influence on nutcracker abundance, serves as the starting point for regional and even broader conservation of whitebark pine, Clark's nutcrackers, and their interaction (Tomback et al., 2022, 2023).

## 2 METHODS

From July 2019 through October 2021, we surveyed five different forest community types for nutcracker visitation in Yellowstone National Park to assess how habitat‐related variables and seasonal timing influence nutcracker abundance during the late summer and fall, when nutcrackers harvest and cache conifer seeds. We also determined through late fall and winter surveys whether nutcrackers were present in park forest communities during winter months. Two forest community types selected for study, lodgepole pine (*Pinus contorta*) and Engelmann spruce (*Picea engelmannii*), have not previously been examined as potential seed resources for Clark's nutcracker. The conifer species included in this study are whitebark pine, limber pine, and lodgepole pine, which have cones that ripen by early September, and Engelmann spruce and Douglas‐fir, which have cones that ripen by early October (Folwells, 1965; Krugman & Jenkinson, 1974).

### 2.1 Yellowstone National Park study area

Yellowstone National Park comprises an area of about 8991 km^2^ or about 10% of the region referred to as the Greater Yellowstone Ecosystem (GYE) (https://www.nps.gov/) (Figure 2), which covers northwestern Wyoming and adjacent southern Montana and eastern Idaho. The park is surrounded by private and public lands comprising the GYE, estimated at about 89,000 km^2^ in area. The public lands are managed by the National Park Service, U.S. Forest Service, U.S. Fish and Wildlife Service, and Bureau of Land Management.

About 83% of the area of Yellowstone National Park is forested (Renkin & Despain, 1992) and ranges in elevation from 1610 to 3462 m (Figure 2) (https://www.nps.gov/; Powell & Hansen, 2007; Renkin & Despain, 1992). Lodgepole pine forms the dominant seral forest community over approximately three quarters of forested areas in the park, with Douglas‐fir, subalpine fir (*Abies lasiocarpa*), Engelmann spruce, and whitebark pine as late seral or climax forest community types (Renkin & Despain, 1992). These conifers are also represented in early seral forest communities along with lodgepole pine. Limber pine occurs on steep slopes and rocky stream banks as solitary trees, in small stands, or as patchy, open, mature stands in the northern region. The five forest communities utilized in this study were dominated by (and named) whitebark pine, limber pine, Douglas‐fir, lodgepole pine, and Engelmann spruce.

### 2.2 Transect selection and study design

Using Yellowstone National Park Habitat Type layers (IRMA database), we identified 25 potential study sites within 3 km of park roads, including the five different forest community types listed above. Our goal was to establish two study sites per forest community type but three study sites in whitebark pine (connected with a future monitoring protocol). In July 2019, we (THM and DFT) ground‐truthed each of the 25 locations to assess whether the designated forest community types were consistent with the habitat types indicated by park GIS layers and, if so, of sufficient area for a 1 km‐long transect. We used GIS to map within each potential study site a circular 1.5 km radius polygon with a 50 m buffer from roads and trails and several randomly generated points for potential transect starts within each polygon. After discarding sites that differed from GIS habitat designation or did not meet habitat requirements, we established eight study sites (including three whitebark pine study sites) in community types dominated by whitebark pine, lodgepole pine, Douglas‐fir, and limber pine. In July 2020, we followed the same protocol to identify a second study site in a limber pine community, which we had discovered in 2019, and two study sites in Engelmann spruce communities, for a total of 11 study sites. At each of these 11 study sites, we established a virtual 1 km transect using a randomly generated start point. Transects generally followed elevational isoclines and stretches of continuous forest habitat. We avoided roads and designated trails at all study sites except for Mammoth Campground. This study site is within a mature limber pine community that encompasses the campground and park headquarters. Given the scarcity of large limber pine stands, we included this study site and found that disturbance did not preclude nutcracker visitation. Along each transect, we tagged 11 trees of the site's dominant conifer species for annual counts of unripe cones. Cone count trees were spaced about every 100 m on or near each transect. Only mature trees with evidence of prior cone production were selected for cone counts.

### 2.3 Transect surveys

We established five point count stations along each virtual transect, spaced 250 m apart. Two people performed each point count survey—one recorder and one observer (Krementz et al., 1997). Each point count was preceded by a quiet 2‐min “settling period” followed by a 15 min nutcracker count session, separated into three 5‐min time intervals, following a time‐removal design (Farnsworth et al., 2002). The observer counted each Clark's nutcracker sighted during a point count and measured the horizontal distance to the bird or tree nearest the bird using a laser range finder (Buckland et al., 2015). The observer and recorder attempted to track previously counted birds as they moved through the forest to avoid recounting the same bird or group. For each visual detection, the observer communicated the bird's behavior, such as flying through the forest, perching quietly, perching vocalizing, seed harvesting, seed caching, and seed cache retrieval. These behavioral observations provided information about how nutcrackers used different forest communities.

Individuals familiar with Clark's nutcracker appearance, vocalizations, and behavior led all transect survey teams. New surveyors received training in nutcracker identification and distance sampling point count methods but recorded data for a week before participating as an observer in surveys. We did not conduct point counts during rain, snow, or high winds, which affect bird activity and reduce detectability. If weather conditions changed rapidly, we paused the survey until conditions improved.

We conducted two early harvest transect surveys of all study sites from 2019 through 2021, which encompassed the dates July 8 through August 11; one mid‐harvest transect survey for all sites, September 2 through September 11; and one late‐harvest transect visit from September 30 through October 13. Heavy, early snow precluded late‐harvest access to one whitebark pine site.

### 2.4 Transect cone count surveys

We estimated cone abundance during the first July visit to each study site by counting cones on each of 11 tagged trees per transect. Two observers counted cones on opposite sides of a tagged tree and summed their counts to achieve a total cone index, a measure of the magnitude of a tree's annual cone production. Cone count stations on opposite sides of each tagged tree (distances and compass directions from each tree) were established when each transect was installed and were used for cone counts across years. In early July, new cones are unripe and not yet removed by squirrels or other seed predators. During each subsequent transect survey, observers estimated cone ripeness for the same six of the 11 transect cone count trees. In cone ripeness surveys, cones of the year were assigned to one or more of the following categories: unripe (scales closed), scales separating, ripe (scales open), or no cones present. Unripe cones required some training to recognize: The unripe cones of spruce, lodgepole, limber pine, and Douglas‐fir cones were distinct from older cones. Whitebark pine cones do not open when ripe; instead, their color changes from dark red‐purple to dark brown (Krugman & Jenkinson, 1974; McCaughey & Tomback, 2001).

### 2.5 Transect‐based community assessment surveys

To describe forest community structure and composition for each transect, we conducted two community assessment surveys, each on a 10 m × 50 m belt transect centered on the virtual transect, beginning at a randomly selected cone‐count tree. Within each belt transect, we counted every living and dead stem that was breast height (1.37 m) or taller, identified it to species, and obtained a diameter at breast height (DBH) in centimeters using a standard forestry DBH tape.

### 2.6 Winter road transect survey

To determine nutcracker presence in park forest communities from late fall through early spring, in November 2019, we established an 80 km‐long roadside survey for nutcrackers across the northern road (the only road open in winter) starting at Mammoth Terrace in the west and ending at Warm Creek Picnic Area, 1.8 km from the northeast park boundary. We selected 10 point count locations along the road, using vehicle pullouts, available parking areas, and the associated forest community type identified by Yellowstone Habitat Type layers. Each point count location was ground‐truthed to confirm forest community type designations. The forest community types included: limber pine/Douglas‐fir (three stops), Douglas‐fir (two stops), lodgepole pine (one stop), subalpine fir (two stops), Engelmann spruce (one stop), and Engelmann spruce/subalpine fir (one stop). GIS layers showed no whitebark pine forest community types near the road.

At each stop, observers performed a 15‐min point count, separated into three 5‐min time intervals, similar to the forest community point counts. Surveys were only performed during days without precipitation or high winds (hand‐held Kestrel 3000; wind speeds below 19 mph). The road surveys were conducted once a month from November through March for 2019–2020 and 2020–2021. Point counts generally followed the same point count protocol used not only on the forest community transects but also counted nutcracker vocalizations as evidence of presence. Distance sampling methods were not used for analysis due to an insufficient number of nutcracker detections at each stop.

### 2.7 Analysis of cone count data

To model how annual cone production varied among conifer species and years, we conducted a regression of “total cones counted per tree” using categorical variables for forest community type and year. Each sample unit was the total number of new cones counted from one tagged tree during a particular year. The distribution of cone counts indicated strong right‐skewness (Figure S1 in Appendix S1). To address the possible over‐dispersion in “total cones per tree,” we compared Poisson and negative binomial regression models using AICc values, which strongly indicated that the negative binomial model had better predictive capacity. Inspection of residual plots showed no indication of model misspecification.

### 2.8 Hierarchical distance sampling model framework

Counts from point count surveys are frequently limited by imperfect detection (Buckland et al., 2015). Studies in which detectability is not estimated either assume perfect detection or that detectability does not change substantially during the course of the study. To make inferences about Clark's nutcracker abundance and forest community use during the conifer seed harvesting season, we selected a hierarchical distance sampling framework that uses point count data to estimate detectability, availability, and abundance simultaneously (Amundson et al., 2014; Nichols et al., 2009).

Imperfect detection can be conceptualized as “present but undetected” or as “temporarily unavailable for detection” (Nichols et al., 2009). Clark's nutcrackers are highly mobile with large home ranges, and individuals of a mobile species may have a range that only partly overlaps the survey area (Lorenz & Sullivan, 2009). During a point count survey, individual nutcrackers may use a part of their range outside the survey area and be considered “temporarily unavailable.” This process theoretically results in underestimation of the number of individual nutcrackers that use a site. In this study, we recorded distance to each detected individual using a laser range finder and the point count time interval of each detection, which enables us to use these data to account for individuals that are “present but undetected” and “temporarily unavailable for detection” (Amundson et al., 2014; Nichols et al., 2009).

We employed distance sampling techniques to estimate detectability of nutcrackers by modeling the decline in detection frequency at increasing distances from the observer (Buckland et al., 2015). Conventional distance sampling makes four core assumptions regarding the data generating process: Individuals at zero‐distance from the point are detected with certainty, individuals are detected at their original locations, individuals are distributed randomly with respect to the point, and distances to detections are measured accurately. More recent research has shown that several of these assumptions can be relaxed by combining multiple survey methods. For example, Amundson et al. (2014) relaxed the assumption of certain detection at zero‐distance by combining distance sampling and time‐removal methods. The only assumption we were uncertain in meeting is that individuals are detected at their original locations, which implies that during the course of the survey, animal movement is negligible (Buckland, 2006). Simulations have shown that a failure to meet this assumption can result in upward bias in abundance estimates due to a change in the shape of the estimated detection function (Glennie et al., 2015). To investigate whether shorter point count time intervals can eliminate nutcracker movement bias, we compared detection curves for nutcracker count datasets based on the first 5 min, the first 10 min, and the total 15 min (Figures S2 and S3 in Appendix S1). Comparison of estimated detection probability curves and confidence intervals revealed no clear differences among these 3‐point count time lengths (Glennie et al., 2015). Consequently, we used the full 15‐min count data for distance sampling analyses. For analysis, we truncated distant nutcracker detections beyond 125 m (Figure S2 in Appendix S1). We used distance data to model the shape of the detection function (which describes the probability of detection as a function of distance) and estimate the proportion of nutcrackers that went undetected during surveys.

We applied time removal to account for temporary availability during surveys by noting the 5‐min point count interval within which each individual animal was recorded. All individual nutcrackers recorded in preceding time intervals of a point count were not recounted to the best of our ability (Farnsworth et al., 2002). By estimating the rate of decline in new detections, we can infer the proportion of individuals that was temporarily unavailable for detection during surveys (Amundson et al., 2014; Farnsworth et al., 2002).

Hierarchical distance sampling (HDS) models estimate the effects of predictor variables on detectability, availability, and the ecological processes potentially impacting abundance (Chandler et al., 2011; Sillett et al., 2012). To examine nutcracker forest community use across multiple visits, we selected the generalized 3‐level hierarchical distance sampling model of Chandler et al. (2011), where *M*_*i*_ represents the total number of nutcrackers that could be detected during a transect survey over the survey duration at site *i*. *N*_*it*_ represents the number of nutcrackers, among the *M*_*i*_, that are available during a survey occasion, *t*. ***Y***_*it*_ is a vector of distance class‐specific nutcracker counts made at site *i* on survey occasion *t* that arise conditional on *N*_*it*_. *M*_*i*_ is represented as a random variable that is typically modeled as either Poisson‐ or negative binomial‐distributed with mean *λ*:

$$M_{i}\sim \text{Negative Binomial}\;\left(\lambda ,\alpha \right)$$

*N*_*it*_, the number of individuals available to be detected during a survey occasion, can be represented as a draw from a binomial distribution with the number of trials equal to *M*_*i*_ and availability equal to *ϕ*:

$$N_{\mathrm{it}}\sim \text{Binomial}\;\left(M_{i},\phi \right)$$

Last, ***Y***_*it*_ can be represented as a multinomial distance sampling process in which the *N*_*it*_ birds available for detection are observed in one of the distance classes or fall into an unobserved class, and ***π***_*it*_ represents a vector of distance class probabilities (including the unobserved class) computed under a given detection function (half‐normal, hazard rate, or uniform):

$$Y_{\mathrm{it}}\sim \text{Multinomial}\;\left(N_{\mathrm{it}},\pi_{\mathrm{it}}\right)$$

The full likelihood expression for given observations ***Y***_*it*_ is stated in Chandler et al. (2011). Two methods of parameter estimation are maximum likelihood and the Bayesian approach. We use maximum likelihood, as suggested by Chandler et al. (2011), whereby the likelihood function is maximized (after the latent variables *M*_*i*_ and *N*_*it*_ have been integrated out). From the resulting parameter estimates $\hat{\lambda }$ and $\hat{\phi }$, the nutcracker density over a study area *A* is estimated by

$$\hat{D}=\frac{\hat{\lambda }\hat{\phi }}{A}$$

The abundance and availability parameters (*λ* and *ϕ*) and detection function can each be modeled as functions of covariates, resulting in covariate‐dependent nutcracker density estimates. The covariates we considered are listed in Table 1. Time removal was modeled via a 5‐min interval indicator covariate for the availability parameter, whereby availability was allowed to change over 5‐min intervals within each 15‐min point count.

**TABLE 1:** Submodels and explanatory variables used for Hierarchical Distance Sampling model comparison (abundance, detection, or availability), and the corresponding description of each variable.

(A) Nutcracker abundance	
Variables	Description
Forest community type	Categorical nominal variable, indicating major conifer species for each study site.
Survey time period	Categorical nominal variable describing the time period during the cone harvesting season in which the survey was performed. Used as a proxy for conifer cone ripeness.
Survey year	Categorical nominal variable indicating the year the survey was conducted.
Cone production energy index (kJ)	Numeric continuous variable indicating the sum of cone counts from the study site multiplied by the estimated energetic content (see seed energetics table for additional information).
Site ID	Categorical nominal variable with unique ID for each site. For comparison with model of forest community type.

(B) Nutcracker detectability	
Variables	Description
DBH stems	Numeric discrete variable indicating the density DBH stems (per 500 m^2^) measured from two community assessment plots for each site.
Observer ID	Categorical variable indicating the team of observers who performed each survey.
Survey time period	Categorical nominal variable describing the time period during the cone harvesting season in which the survey was performed.

(C) Nutcracker availability	
Variables	Description
5‐min time interval	Categorical nominal variable, representing the time interval during which each nutcracker was detected. For time‐removal survey design.

*Note*: Up to three variables were used in each model including forest community type; and only one interaction between parameters was considered. For detection, only a single parameter was included in each competing model. We compared each model to a null model with no detection covariates. For availability, we compared an intercept‐only model to a model with separate estimates of availability during each survey time period.

### 2.9 Ecological modeling approach and model selection

All analyses used the statistical programming language R (R Core Team, 2022). We modeled nutcracker abundance with a single‐season hierarchical distance sampling approach in which repeated visits to sites were assumed to be independent using the *gdistsamp* function in the Unmarked package (Fiske & Chandler, 2011). While a single season model is preferable for estimating static abundance rather than dynamic trends such as annual trends (Ahlering & Merkord, 2016; Linden et al., 2017), we considered year as a categorical covariate to uncover any significant differences in abundance across years (Kéry & Royle, 2020). Given that nutcrackers are highly mobile searching for seed resources, known to travel as far as 32 km in a single flight, there is likely to be high turnover in birds from one visit to the next, making the assumption of independence tenable (Lorenz et al., 2011; Tomback, 1998). Using this modeling framework, we estimated model parameters, model‐based predictions of abundance, and 95% confidence intervals around predicted values.

We calculated that there were more than 800 possible combinations of variables in our model set including null models (Table 1). We followed a secondary candidate set approach to model identification and selection during analysis (Morin et al., 2020), which involved fitting submodels for each major process of the model: abundance (*M*_*i*_ distribution), detectability, and availability. To assess model goodness of fit, we performed a parametric bootstrapping procedure (Kéry & Royle, 2015), where we estimated goodness of fit metrics including sum of squared errors, chi‐square goodness of fit, and Freeman Tukey goodness of fit statistics from data simulated under the top fitting model, using the *parboot* function in the Unmarked package with 500 simulated bootstrap samples (Fiske & Chandler, 2011). We then compared our observed fit statistics to the Kéry and Royle (2015) simulated distributions to assess whether our observed metrics of fit were comparable to those derived from model‐based simulations.

### 2.10 Predictive variables for forest community type use

Our primary study aim was to compare predicted nutcracker density among predefined forest community types found within Yellowstone National Park. The magnitude of nutcracker forest community use during the late summer seed harvesting and caching period was previously related to metrics of seed resources and abundance (Barringer et al., 2012; Ray et al., 2020; Williams et al., 2020).

While foraging for conifer seeds, nutcrackers demonstrate sensitivity to energy availability from seed resources (e.g., Tomback, 1978; Vander Wall, 1988). To investigate the effect of variation in seed energy availability on nutcracker abundance, we calculated a cone production energy index, an estimate of the magnitude of energy available in the annual cone crop of a site. This was done by multiplying the total cone index values, defined above, by estimates of kilojoules per cone for each conifer species. We estimated kilojoules per cone by obtaining data from the literature (Table 2) and performing log transformations because values were highly right‐skewed.

**TABLE 2:** Calculations of food energy per cone using data from the literature for estimates for each of the five conifer species included in our study.

Conifer species	Mean seeds per cone	Standard deviation (SD) in seeds	Mean kilojoules (kJ) per seed	Kilojoules (kJ) per cone
*P. albicaulis*	75^6^	28^6^	2.58^1^	193.50
*P. contorta*	57^1^	18.5^2^	0.07^2^	3.99
*P. engelmannii*	182^2^	25.9^2^	0.06^2^	10.92
*P. flexilis*	93^3^	30.75^5^	2.80^4^	252.00
*P. menziesii*	45^2^	8.2^2^	0.24^2^	10.80

*Note*: Standard deviation in limber pine seed number per cone calculated based on Hozo et al. (2005) using minimum and maximum sample values. Literature sources include ^4^Lanner (1996) and ^5^Hozo et al. (2005), ^2^Smith (1970), ^1^Tomback (1982), ^3^Vander Wall and Balda (1977), and ^6^Weaver and Forcella (1986).

Given that we stratified study sites by forest community type a priori, we parameterized the abundance submodel with forest community type covariates (Table 1). We posited that during the seed harvest period, model‐based predictions of higher nutcracker density would indicate forest community preference and likely seed resource use. Given that our second study question was to investigate how site and survey level variables associated with conifer cone abundance might influence predictions of nutcracker density during cone harvest season (Table 1), we also investigated the effects of survey date, year, and cone production energy index on predicted nutcracker density, by including them as abundance covariates. We predicted that nutcracker abundance would be highest during the time period when whitebark pine and limber pine cones were ripening and lower when the cones were unripe or the cone crops were depleted. We also expected that nutcracker abundance would be related to the size of the annual cone crop for conifer species which were used as seed resources, and thus we expected a positive relationship with our index of cone production energetics for conifers that are potential seed resources for nutcrackers. Support for these effects on nutcracker density would indicate preference for forest community types with abundant and energetically efficient seed resources, which would be consistent with predictions from foraging theory and previous nutcracker studies (Stephens & Krebs, 2019; Tomback, 1978; Vander Wall, 1988; Williams et al., 2020).

## 3 RESULTS

### 3.1 Cone ripening phenology and nutcracker observations

Nutcracker point count surveys were conducted during early‐, mid‐, and late‐harvest time periods which corresponded with the different stages of cone ripeness for the conifer species considered in this study. During July and early August (early time period), cones for all conifer species in our study were unripe. In late August and early September (mid‐time period), whitebark pine and limber pine cones began to ripen followed closely by cones of lodgepole pine. In mid‐ to late September (late time period), Douglas‐fir and Engelmann spruce cones began to ripen.

In July and early August, nutcrackers harvested the unripe seeds from whitebark pine cones (Table S1 in Appendix S2). As these cones ripened in late August and September, we observed higher counts of nutcrackers in whitebark forest communities (Figure S4 in Appendix S1). Nutcracker counts in whitebark pine forest communities declined slightly in October, because cones were depleted. Similarly, in limber pine forest communities, we observed an increase in nutcracker counts during September, when cones were opening, and a decrease in October after cones were depleted. Nutcracker counts in lodgepole pine and Engelmann spruce communities were generally low with a somewhat higher count in Douglas‐fir communities during early‐ and late‐harvest time periods (Figures S4 and S5 in Appendix S1).

### 3.2 Harvesting and seed caching observations

We observed 46 instances of nutcrackers harvesting seeds from cones during our surveys. Due to unequal survey effort among forest types, we summarized these observations (Table S1 in Appendix S2) as an average per survey. Eighty‐seven percent of harvesting observations occurred in whitebark pine and limber pine forest community study sites, but we also observed nutcrackers harvesting seeds from lodgepole pine and Douglas‐fir cones. Despite an abundance of cones in some years, Engelmann spruce was the only conifer species we did not see used by nutcrackers as a seed resource.

We observed seed caching behavior 10 times during our point count surveys (Table S1 in Appendix S2): three of these observations occurred in whitebark pine communities, one in an Engelmann spruce community, five in limber pine communities, and one in a Douglas‐fir community. No caching behavior was observed during lodgepole pine point count surveys. However, in 2021, we observed three instances of lodgepole seed harvesting. In some cases, seed harvesting and caching behaviors occurred in rapid succession, making it obvious which conifer seeds were cached. In several instances, which conifer seeds were being cached was unknown. During September 2020 and 2021 at our Dunraven study site, but off‐transect, we (DFT) observed many nutcrackers fill their throat pouches with whitebark pine seeds. Many of these birds subsequently flew downslope in the direction of lower elevation lodgepole pine communities or toward the Grand Canyon of the Yellowstone, which has steep, rocky walls, presumably to cache the seeds.

### 3.3 Hierarchical distance sampling model

AICc scores strongly supported selection of a negative binomial distribution for nutcracker abundance (*M*_*i*_). Thus, all subsequent models were based on negative binomial regression. We found that 15 abundance submodels had a Delta‐AICc of <10, including the Null model and the Site ID‐only model. Comparison of submodels for the nutcracker detection function indicated that DBH stem density was an important factor in nutcracker detection. The half‐normal and hazard rate shape functions for detection were advanced to the final candidate set of models; no other detection submodels had ∆AICc <10. The submodel that incorporated differences in nutcracker availability among 5‐min time intervals was strongly favored over a constant availability submodel. Submodels with ∆AICc <10 were advanced to the final candidate set of models (Morin et al., 2020).

For the final stage of model selection, using the secondary candidate set approach, we evaluated all combinations of detection and abundance submodels (Table 3). The ΔAICc = 0 model included abundance covariates for each forest community type and survey time period, availability covariates for 5‐min time interval, and a single detection covariate for DBH stem density (Table 4). This model had approximately 49% of the AICc weight in the model set, whereas the next closest model was similar but did not contain abundance covariates for survey time period (ΔAICc = 1.3). Together, these two models included approximately 74% of the AICc weight in the model set. Results of the bootstrap procedure for the sum of squares errors, chi‐square goodness of fit, and Freeman–Tukey test statistics are presented in the supporting documentation (Table S2 in Appendix S2).

**TABLE 3:** Comparison of final Clark's nutcracker forest resource use models.

Model names	*K*	AICc	Δ AICc	Model Lik	AICc Wt	LL	Cum. Wt
~Forest type + Month	13	778.944	0	1	0.488	−374.556	0.488
~5‐min time interval							
~Stems ~Half normal							
~Forest type	11	780.245	1.301	0.522	0.254	−377.761	0.742
~5‐min time interval							
~Stems ~Half normal							
~Forest type + Energetics index	12	782.52	3.576	0.167	0.082	−377.635	0.824
~5‐min time interval							
~Stems ~Half normal							
~Forest type + Month + Year	15	782.593	3.649	0.161	0.079	−373.716	0.902
~5‐min time interval							
~Stems ~Half normal							
~Forest type + Year	13	783.337	4.393	0.111	0.054	−376.753	0.956
~5‐min time interval							
~Stems ~Half normal							
~Forest type + Month	14	785.93	6.986	0.03	0.015	−376.731	0.971
~5‐min time interval							
~Stems ~Hazard‐rate							
~Forest type + Month * Energetics index	16	786.158	7.214	0.027	0.013	−374.123	0.985
~5‐min time interval							
~Stems ~Half normal							
~Forest type	12	787.016	8.072	0.018	0.009	−379.883	0.993
~5‐min time interval							
~Stems ~Hazard‐rate							
~Forest type + Energetics index	13	789.325	10.381	0.006	0.003	−379.747	0.996
~5‐min time interval							
~Stems ~Hazard‐rate							
~Forest type + Month + Year	16	789.702	10.758	0.005	0.002	−375.895	0.998
~5‐min time interval							
~Stems ~Hazard‐rate							
~Forest type + Year	14	790.222	11.278	0.004	0.002	−378.877	1
~5‐min time interval							
~Stems ~Hazard‐rate							
~Forest type + Month * Energetics index	17	795.33	16.386	0	0	−377.302	1
~5‐min time interval							
~Stems ~Hazard‐rate							

*Note*: The first component in the model name represents the nutcracker abundance submodel, the second component the availability submodel, the third component the detection submodel, and the fourth component the detection function shape. All models have a negative binomial distribution for the abundance process (*M*_*i*_). For each model, AICc and ΔAICc values, the number of parameters (*K*), a ratio of the model likelihood relative to that of the lowest AICc model (Model Lik, computed as *e* ^−ΔAICc/2^), the AICc weight (AICc Wt), log likelihood (LL), and cumulative AICc weight (Cum. Wt) are provided.

**TABLE 4:** Parameter estimates for the ΔAICc = 0 model for Clark's nutcracker abundance (A), nutcracker detectability (B), nutcracker availability (C), and negative binomial dispersion (D).

(A) Abundance				
Parameters	Estimate	SE	*z*	*p*(>|*z*|)
PIAL (intercept)	−0.69919	0.317745	−2.20046	.027774
PICO	−1.98792	0.478559	−4.15398	3.27E‐05
PIEN	−2.16405	0.556419	−3.88925	.000101
PIFL	−1.85267	0.427752	−4.33117	1.48E‐05
PSME	−2.26249	0.427265	−5.29528	1.19E‐07
Mid‐harvest	0.846222	0.343648	2.462464	.013799
Late‐harvest	0.570917	0.381871	1.495051	.134901

(B) Detectability				
Parameters	Estimate	SE	*z*	*p*(>|*z*|)
Intercept	3.916925	0.079672	49.16298	0
DBH stems	−0.00753	0.000977	−7.70518	1.31E‐14

(C) Availability				
Parameters	Estimate	SE	*z*	*p*(>|*z*|)
Intercept	1.59576	1.396909	1.142351	.253308
Time interval 2	−2.00326	1.111411	−1.80245	.071475
Time interval 3	−1.55436	1.04932	−1.48131	.138525

(D) Negative binomial dispersion				
Parameters	Estimate	SE	*z*	*p*(>|*z*|)
Intercept	−0.22292	0.260189	−0.85677	.391574

*Note*: See Table 3 for model name and parameters. Abundance intercept is defined as whitebark pine forest habitat during early‐harvest time period. Mid‐harvest and late‐harvest parameters are offsets for the corresponding harvesting time periods. Availability intercept is defined as the first 5‐min time period during 15‐min point counts. Parameters for Time interval 2 and Time interval 3 represent offsets for corresponding 5‐min time intervals.

Abbreviations: DBH, diameter at breast height; PIAL, whitebark pine; PICO, lodgepole pine; PIEN, Engelmann spruce; PIFL, limber pine; PSME, Douglas‐fir.

**FIGURE 3:**
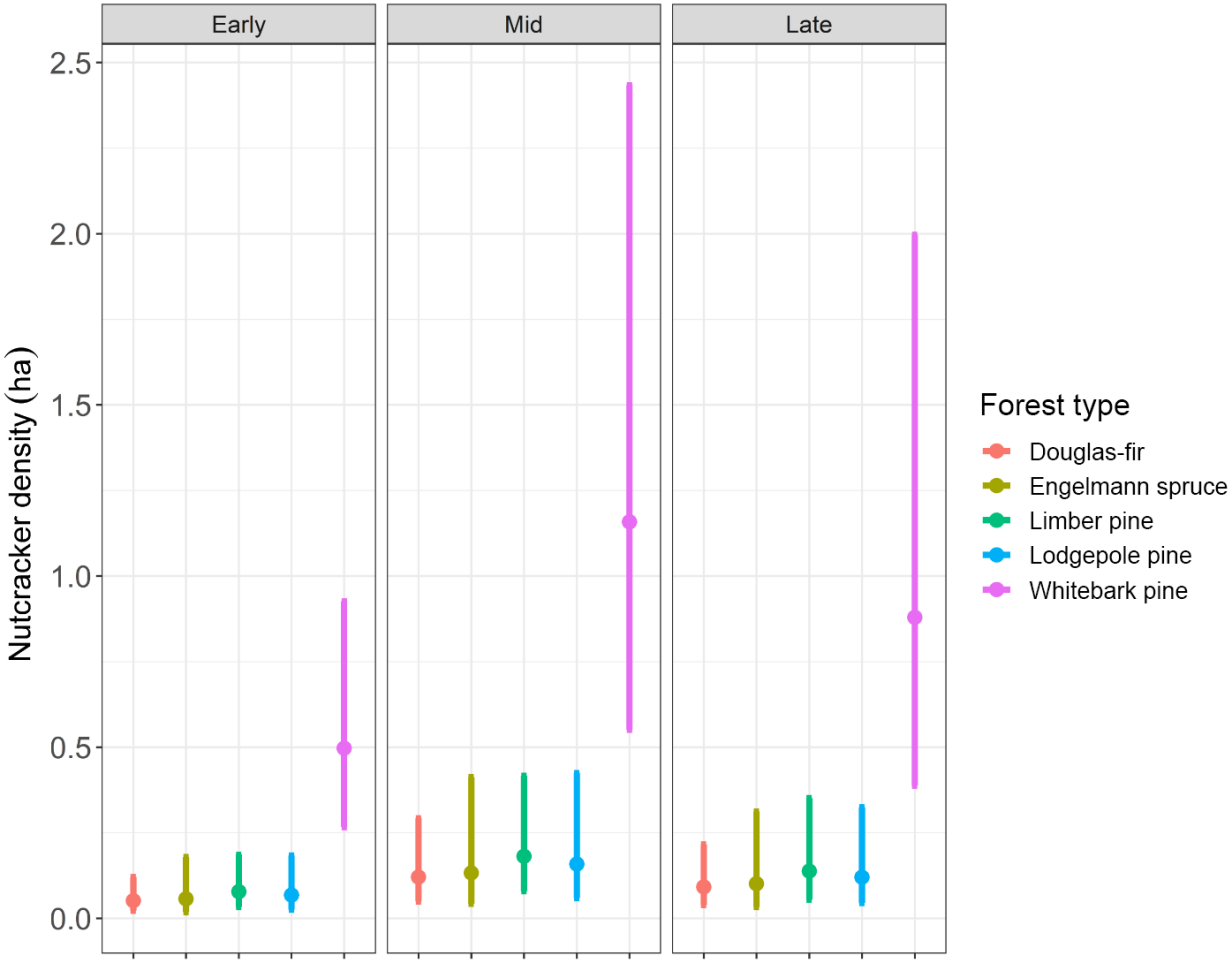
Prediction plot of average nutcracker density within each forest community type within our study during the three survey periods. Early‐, mid‐, and late‐harvesting time periods are presented from left to right. Intervals represent 95% confidence intervals around each estimate. Nutcracker density confidence intervals for whitebark pine did not overlap with confidence intervals for other forest types, whereas confidence intervals for all other forest types overlapped with one another.

**FIGURE 4:**
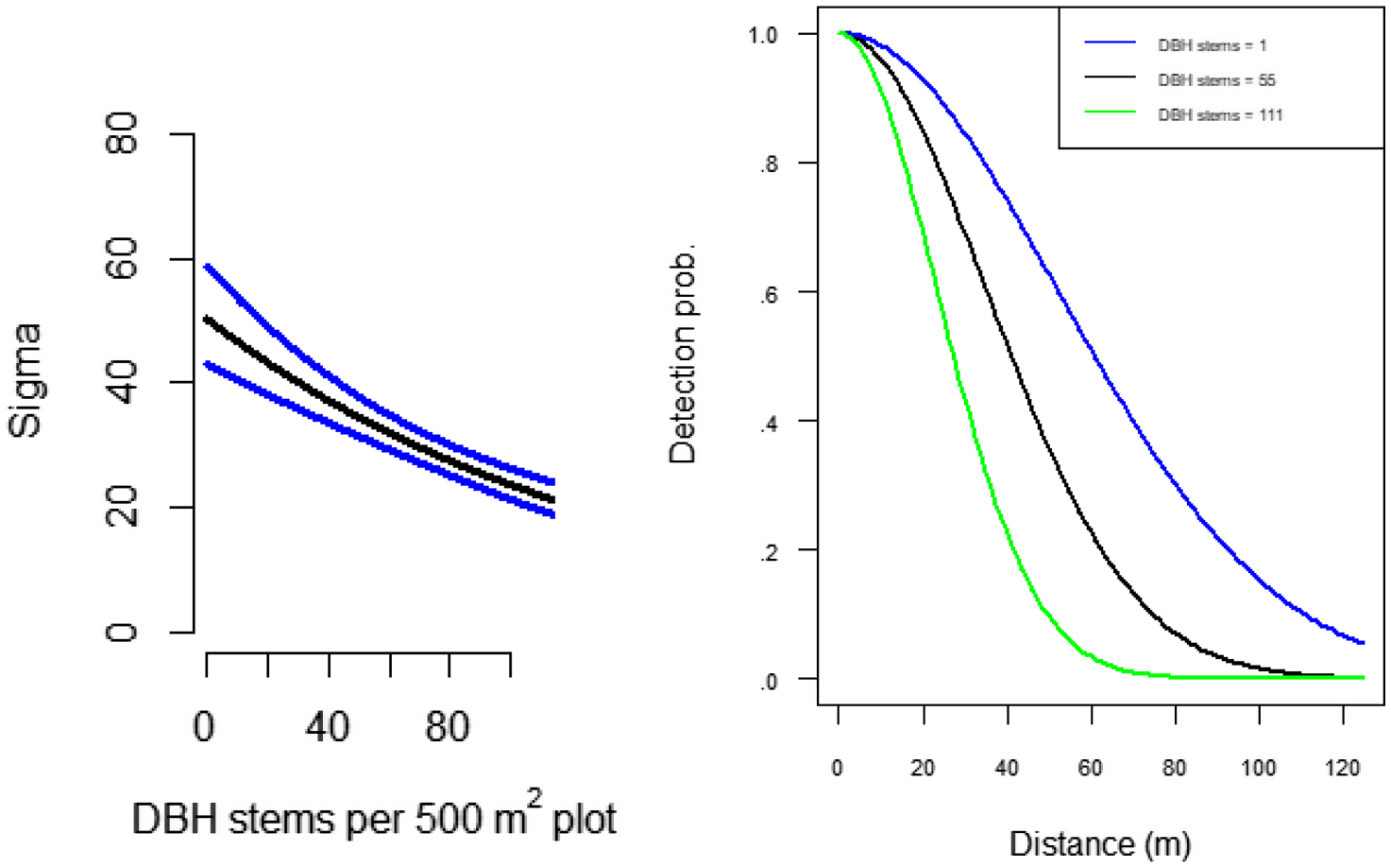
Left: Detection function scale parameter in response to the density of DBH stems (per 500 m^2^) from community assessment surveys. Blue lines indicate 95% confidence intervals. Smaller values of sigma, the scale parameter for the half‐normal detection function, result in a lower detectability estimate for nutcrackers. Right: Estimated detection probability at minimum, midpoint, and maximum stem density values.

Model‐based predictions and 95% confidence intervals are presented in Figure 3. We found that the half‐normal detection function had a smaller AICc value than the hazard rate detection function. We also found a negative relationship between nutcracker detectability and DBH stem density (Figure 4, Table S3 in Appendix S2). Clark's nutcracker density was highest in whitebark pine forest community sites. We found that predicted nutcracker density was lowest in our early harvesting season surveys and highest during our mid‐harvesting season surveys. Aside from whitebark pine, confidence intervals for predicted nutcracker density among other forest community types overlapped with one another, indicating no discernable difference in predicted nutcracker density among them.

### 3.4 Cone count analysis and energetics

**FIGURE 5:**
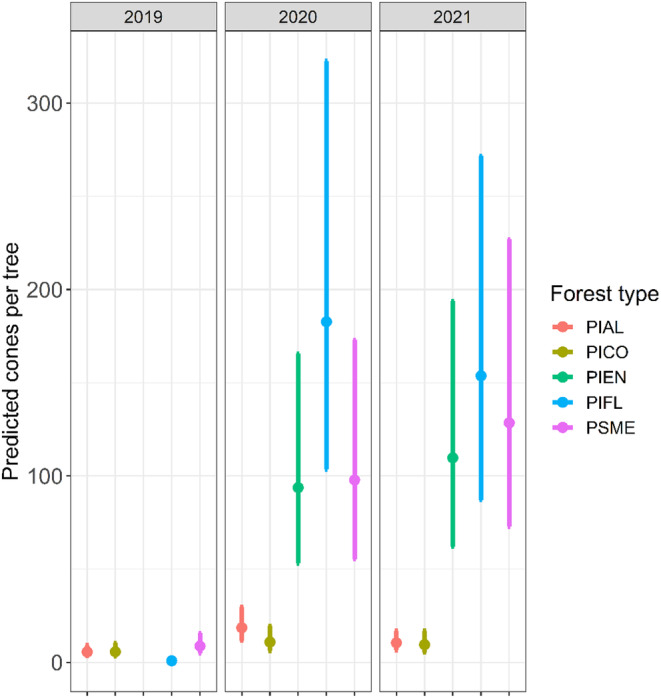
Model‐based predictions from negative binomial regression of cone count data. PIAL, whitebark pine; PICO, lodgepole pine; PIEN, Engelmann spruce; PIFL, limber pine; PSME, Douglas‐fir. Intervals represent 95% confidence intervals around predicted values. Whitebark pine and lodgepole pine had relatively few cones per tree in comparison to Engelmann spruce, limber pine, and Douglas‐fir. For all conifer species, cones per tree were estimated as lower in 2019 than in 2020 and 2021. Engelmann spruce study sites were first established in 2020, and as a result, there are no cone count data for Engelmann spruce for 2019.

Information on seed energetic values came from different literature sources, seed collections from regions outside the GYE, and different laboratory methods; the information may only be used for a very general comparison (Table 2), because conifer seed characteristics often vary geographically (e.g., Benkman, 1995; Cwynar & MacDonald, 1987). The highest energetic values for both cones and seeds are for limber pine and whitebark pine. Engelmann spruce has the lowest mean energy content per seed, with each seed containing about 0.02 the value of a limber pine and whitebark pine seed. Lodgepole pine cones, however, have the lowest mean seed energy value per cone, representing about 2% of the seed energy value of a limber pine or whitebark pine cone. Douglas‐fir seeds have higher energetic value than lodgepole pine or spruce seeds, but the higher seed number in spruce cones results in higher seed value per cone. Initial data investigation indicated overdispersion in cone counts and large differences in counts among conifer species (Figure S1 in Appendix S1). AICc values favored negative binomial regression over Poisson regression. We compared all cone count models to an “intercept only” model and found that the best supported model included the variables *conifer species* and *year*, as well as an interaction between the two. This model provides evidence for differences in mean number of cones per tree among conifer species in our study (Figure 5). Additionally, the interaction between conifer species and year indicates that interannual changes in predicted numbers of cones per tree differ among conifer species. The smallest predicted number of cones per tree occurred in 2019 for all conifer species. In 2020 and 2021, Engelmann spruce, limber pine, and Douglas‐fir produced greater numbers of cones per tree than in 2019 and had more variation in cones per tree than whitebark pine and lodgepole pine. Across all years, whitebark pine and lodgepole pine had lower predicted numbers of cones than Engelmann spruce and Douglas‐fir; limber pine showed high variability across years. For whitebark pine, lodgepole pine, and limber pine, the largest number of predicted cones per tree occurred in 2020. For Engelmann spruce and Douglas fir, the largest number of cones per tree occurred in 2021. However, despite relatively high cone production in Engelmann spruce and Douglas‐fir in 2020 and 2021, these forest community types did not attract high nutcracker densities during the fall seed harvest period (Figure 3, Figure S4 in Appendix S1).

### 3.5 Winter road surveys and cache retrieval

Winter road surveys were originally implemented to determine whether nutcrackers used the park in late fall and winter months, and, if so, which forest communities they might occur in. We determined that there were too few nutcracker detections from winter road surveys to conduct a distance sampling analysis, but the results were instructive.

During the 2019–2020 period, we performed a total of 50 point counts on the roadside transect, and for the 2020–2021 period, 40 point counts (Table S4 in Appendix S2). For the 2019–2020 period, we saw or heard nutcrackers on 22% of point counts; for the 2020–2021 period, we saw or heard nutcrackers on 52% of point counts. Overall, we saw or heard nutcrackers during 30% of the limber pine/Douglas‐fir point counts, 38% of Douglas‐fir counts, 33% of lodgepole pine counts, 33% of subalpine fir counts, 56% of Engelmann spruce counts, and 33% of Engelmann spruce/subalpine fir counts. The road transect point counts indicated that nutcrackers were present in all road transect forest community types during the late fall and winter seasons (Table S2 in Appendix S2).

We observed nutcrackers retrieve seed caches from rocky outcroppings in the Douglas‐fir community near a point count transect in mid‐March in both 2019 and 2020, indicating their use of seed caches within this forest community during the breeding season.

## 4 DISCUSSION

In this study, we addressed questions concerning the forest community types used by Clark's nutcrackers within Yellowstone National Park during seed harvest and caching; whether some nutcrackers remain in the park during late fall and winter; how survey timing, cone ripeness, and cone production influenced nutcracker habitat use; and whether nutcrackers used seed resources beyond whitebark pine within the park.

Using a hierarchical distance sampling approach, we were able to estimate nutcracker abundance for each conifer forest community type considered in our study. In addition, we compared models to assess whether survey timing, cone ripeness, and cone production index impacted estimates of nutcracker abundance. By recording observations of harvesting and caching during our surveys, we were able to investigate which seed resources nutcrackers used within Yellowstone National Park. Finally, by implementing a road transect survey, we determined whether some nutcrackers used the park during late fall and winter months.

### 4.1 Hierarchical distance sampling model

We found that nutcracker detectability was negatively related to the density of DBH‐sized stems at each study site, likely owing to visual obstruction caused by increasing tree density. We also found that the number of newly observed birds was highest in the first 5‐min interval and lower in the second two intervals, as would be expected under a time‐removal sampling survey design. We interpret our model‐based estimates of availability to represent the probability that a nutcracker that is using the forest community site being surveyed will be within the survey radius during the survey.

### 4.2 Cone count analysis and cone energy indices

Model comparison strongly supported an interaction between cone production index and year. This suggests that cone production per tree varied among conifer species and across years and that higher cone production for different species did not always occur in the same year (Figure 5). While cone production was lowest in 2019 for all conifer species, the magnitude of increase in subsequent years differed among species. Whitebark pine, limber pine, and lodgepole pine all showed higher cone production in 2020 than in 2019 and slight declines in 2021. Conversely, Engelmann spruce and Douglas‐fir showed increases in cone production from 2019 through 2021. The trends in cone production across years may have been influenced by the inclusion of new limber pine and Engelmann spruce forest community study sites during 2020. Estimates for limber pine in 2019 were based on a single site near Mammoth, but in successive years, estimates included a second site in the Lamar Valley.

Model comparison also did not support the use of cone production energy indices in predicting nutcracker abundance during our surveys. While there was, in fact, a positive relationship between cone production energy index and nutcracker abundance, the models which included forest community type and not energy index had lower values of AICc and thus better predicted nutcracker abundance. Due to the high annual variability in both nutcracker abundance and conifer cone production, further study into predictive relationship between these two variables is warranted. In particular, our whitebark pine cone production indices were low to moderate across all 3 years, which agrees with cone count data gathered independently for whitebark pine by the Interagency Grizzly Bear Study Team (e.g., Haroldson, 2019). The lower whitebark pine cone production is counter‐balanced by high energy value per seed and per cone (Table 2). Collecting data on a larger range of annual cone production conditions may help further our understanding of the relationship between nutcracker abundance and cone production; this effort is currently in progress.

### 4.3 Nutcracker abundance and seed resources

Our model results show higher estimated nutcracker abundance in whitebark pine forest communities compared to the other forest community types included in this study, supporting the prediction that whitebark forest communities are the most important seed resource for Clark's nutcracker. In addition, we found that estimated nutcracker abundance increased overall during the mid‐harvesting survey period when cones of whitebark pine are ripe. In addition to having the highest cone and seed energy, whitebark pine seeds ripened several weeks earlier than those of other conifers, with the exception of limber pine, providing food after previous years' caches had been retrieved (Table 2) (e.g., Tomback, 1998; Tomback, 1978). Whereas this trend in nutcracker abundance is likely to be driven by results from surveys in whitebark forest communities, an interaction between forest community type and survey time period was ranked lower by AICc value. Nutcracker abundance was found to be lowest in the early‐harvest survey period before the cones of any conifer species ripened. During the late‐harvest survey period, we found that nutcracker abundance was intermediate. Our model results and harvesting observations indicate that within Yellowstone National Park, whitebark pine represents a primary food resource for Clark's nutcracker.

Despite a relatively high number of nutcracker observations in limber pine, which also has earlier ripening, energy‐rich seeds, predicted nutcracker density was not discernably different than that modeled for lodgepole pine, Engelmann spruce, or Douglas‐fir communities. This is likely due in part to the effect of stem density on nutcracker detectability. The limber pine stands were relatively open, resulting in a low stem density and thus a relatively high estimated nutcracker detectability. Thus, surveyors in limber pine study sites may have detected a higher proportion of the birds present in comparison to other forest community sites with denser tree composition. In addition, nutcracker activity in limber pine was observed during the mid‐harvesting survey period when limber pine cones were ripe, that is, scales opening. Conversely, during early harvesting periods, limber pine cones are closed, very resinous, and less energetically efficient for nutcracker harvest if other foods are available. During the late‐harvest season survey period (October), limber pine cones are depleted of seeds, and the majority of nutcrackers have moved on (Williams et al., 2020).

Our harvesting observations supported the use of limber pine as a seed resource by some nutcrackers, including family groups. Of note, nutcrackers harvested and cached limber pine seeds in Yellowstone National Park at the same time that relatively high numbers of nutcrackers were present in the more widely distributed whitebark pine stands in the park. Given the energetic equivalency between whitebark pine and limber pine seeds (Table 2), the decision by some birds to use this seed resource may avoid competition for seeds in whitebark pine stands. Because limber pine cones open and whitebark pine cones do not, mature seeds drop from limber pine cones and are likely consumed by other birds and mammals, reducing foraging efficiency later in September by nutcrackers who continue to sort through limber pine cones for seeds. By the time we conducted our October point counts each year, nutcrackers were no longer harvesting limber pine seeds but continued to harvest whitebark pine seeds in at least one of the whitebark pine study sites.

Although we observed nutcrackers harvesting limber pine seeds frequently during our surveys, the importance of limber pine as a seed resource for nutcrackers in Yellowstone National Park requires further study. The few stands of healthy limber pine in the GYE and scattered patches along river banks in the northern Yellowstone National Park region may be important nutcracker seed resources. Limber pine, however, is declining from the same threats as whitebark pine. In addition, many cone‐bearing limber pine trees in our study site in the Lamar Valley have been damaged extensively by American bison (*Bison bison*) horn‐rubbing (D. F. Tomback, T. H. McLaren, W. Wehtje, L. E. Walker, D. W. Smith, unpublished observations). Limber pine is patchily distributed across a broad elevational range in the montane West and is an important seed resource for Clark's nutcrackers, especially where whitebark pine is absent (e.g., Benkman et al., 1984; Vander Wall, 1988; Williams et al., 2020).

Our analysis of nutcracker abundance indicates that whitebark pine forest community types are the most important resource for nutcrackers during the fall conifer seed harvesting season within Yellowstone National Park and likely across the GYE. The lower abundance of nutcrackers in other forest community types suggests that these other seed resources are less used despite good cone production in some years. As a caveat, we note that these alternative seed resources might be more important in years when whitebark pine cone production fails or is very low; during this study, we had low to moderate whitebark cone production in at least two of our whitebark pine study sites each year. Without widely distributed stands of more preferred alternative seed resources, such as those found in the Northern Cascades, Sierra Nevada, and Rocky Mountain National Park, whitebark pine becomes an especially critical resource for nutcrackers (Figure 1 and references therein) and the primary predictor of nutcracker abundance in the GYE. The ongoing decline in whitebark pine from WPBR may result in progressive loss of carrying capacity for Clark's nutcrackers and in disruption of breeding activity due to insufficient numbers of seed caches (e.g., Barringer et al., 2012; Schaming, 2016).

Given whitebark pine's relatively wide distribution in Yellowstone National Park and the GYE and the comparatively low use of other seed resources regionally whitebark pine appears to be the most important influence on nutcracker carrying capacity. This would suggest that annual whitebark pine cone production levels may strongly influence Clark's nutcracker abundance and population dynamics in the GYE. We hypothesize that the extent and frequency of nutcracker annual movements may be related to the availability of energetically important additional seed resources, such as ponderosa pine or the pinyon pines (Tomback, 1978; Vander Wall, 1988; Williams et al., 2020). In regions with limited seed resources, nutcrackers may wander more widely during seed harvesting season in search of available seeds. Thus, annual nutcracker abundance, reproduction, and movement may be linked more directly to annual whitebark pine cone production in regions where additional seed resources are sparse or nonexistent. Further study of these processes may aid in conserving the coevolved mutualism between these two species by identifying specific conditions in which this relationship is particularly vulnerable to disruption.

We compare our nutcracker abundance results in whitebark pine communities to those of Ray et al. (2020), a study which evaluated the effect of several covariates related to nutcracker seed resource use in a Bayesian hierarchical distance sampling framework. Despite several differences in data collection, including study region, survey timing (month of year and time of day), survey time length, and site revisit schedule, our estimates of predicted nutcracker abundance were comparable. We observed that our model‐based 95% confidence intervals of nutcracker density in whitebark pine forest communities (Early‐harvest: 0.3–1.0 CLNU/ha, mid‐harvest: 0.6–2.4 CLNU/ha, late‐harvest: 0.4–2.0 CLNU/ha) overlapped with the density predictions for sites with high whitebark cover in Ray et al. (2020) for Sequoia‐King's Canyon (0.6–2.0 CLNU/ha) and Yosemite National Parks (0.6–0.8 CLNU/ha). Importantly, the top performing model in our analysis included parameters for harvesting season time‐period. While this complicates any direct comparison of nutcracker density between the two studies, we would suggest that there is reasonable agreement between our abundance estimates.

### 4.4 Nutcracker behavioral observations

While behavioral observations were a secondary priority during our point count surveys, the data support the important role of whitebark in Clark's nutcracker seed resource use. The majority of seed harvesting observations occurred during the early and mid‐harvesting season, when whitebark pine seeds were ripening or ripe. Whitebark pine seeds, which are harvested in late summer or early fall, have not completely developed at the start of our annual transect surveys, but nutcrackers often harvest ripening seeds to feed juveniles (Tomback, 1978). By late August and early September, whitebark and limber pine cones begin to reach full maturity, and nutcrackers harvest and cache seeds. Nutcrackers continued to harvest whitebark pine seeds after cones fully matured. During 2020, we observed nutcrackers harvesting whitebark pine seeds in October, which corresponds to observations by Tomback (1978).

In 2021, we observed nutcrackers harvesting lodgepole pine seeds on three separate occasions, which has not been previously reported except anecdotally (D. F. Tomback, T. H. McLaren, W. Wehtje, L. E. Walker, D. W. Smith, unpublished observations, cited in Lanner, 1998). These observations occurred in the early‐ and late‐harvest periods, indicating that nutcrackers may harvest lodgepole pine seeds when preferred seed resources are either unripe or depleted. We also observed nutcrackers harvesting and caching Douglas‐fir seeds on two occasions and retrieval of Douglas‐fir seeds from caches in late winter and early spring during the breeding period. The late season harvest of Douglas‐fir seeds suggests transition from preferred seed resources as they are depleted. This pattern of use is consistent with observations made by Williams et al. (2020), who documented both harvesting and caching of Douglas‐fir seeds. Williams et al. (2020) and Schaming (2016) suggested that nutcrackers also use Douglas‐fir stands for shelter.

Our ability to document behavior is limited by imperfect detectability, and our distance sampling methods may have prevented us from making more thorough behavioral observations. Thus, these results should be considered chance observations and not true rates of these behaviors.

### 4.5 Winter road surveys

Observations from the winter road surveys indicate that nutcrackers were present within the park during the winter seasons of 2019–2020 and 2020–2021. We observed nutcrackers during all months in which surveys were conducted and in all forest community types that were available along the road transect. These observations indicate that at least some nutcrackers remain in the park after peak harvesting season has passed. Questions remain concerning what proportion of the nutcracker population overwinters in the park and the breeding success of these individuals. We hope to address these questions over time through spatial tracking of individual birds.

## 5 CONCLUSIONS

Our study confirms the importance of whitebark pine for Clark's nutcrackers as a seed resource in Yellowstone National Park. In addition, limber pine, despite its patchy distribution, dependably serves as a seed resource for nutcrackers. Limited use of other, less energetically rewarding seed resources may be a result of the relatively stable whitebark pine cone production during the 3 years in which we completed our surveys or an accurate reflection of nutcracker seed preferences. However, different patterns of nutcracker habitat use could emerge in a year with little to no cone production in whitebark pine and high cone production in other seed resources and especially Douglas‐fir (Williams et al., 2020).

Our findings suggest that whitebark pine is key to maintaining nutcracker populations within Yellowstone National Park. Ray et al. (2020) also found that nutcracker density was positively related to whitebark pine cover in Sequoia‐Kings Canyon and Yosemite National Parks, a region where other seed resources were available. We hypothesize that whitebark pine is highly important not only as a seed resource in the GYE but also key to maintaining nutcracker populations in geographic regions where other seed resources are limited in availability. Threats to whitebark pine forests, especially white pine blister rust and recent large‐scale mountain pine beetle outbreaks, lead to a downward spiral of both birds and whitebark pine regeneration, as observed in areas such as Glacier and Mt. Rainier national parks (Barringer et al., 2012; Ray et al., 2020; Shanahan et al., 2016; Shepherd et al., 2018; Smith et al., 2011). While whitebark pine health has been monitored within the Greater Yellowstone Ecosystem for nearly two decades (e.g., Shanahan et al., 2016), our work has initiated a monitoring effort for Clark's nutcracker in Yellowstone National Park. Continued efforts to monitor nutcracker abundance and whitebark pine health will provide support for timely management intervention to prevent the loss of whitebark pine, Clark's nutcracker, and their iconic interaction.

## AUTHOR CONTRIBUTIONS

**Thomas H. McLaren:** Data curation (equal); formal analysis (lead); investigation (equal); methodology (supporting); project administration (supporting); resources (supporting); software (lead); supervision (supporting); validation (lead); visualization (lead); writing – original draft (lead); writing – review and editing (equal). **Diana F. Tomback:** Conceptualization (lead); data curation (equal); formal analysis (supporting); funding acquisition (lead); investigation (lead); methodology (lead); project administration (lead); resources (lead); supervision (lead); validation (supporting); writing – original draft (supporting); writing – review and editing (equal). **Nels Grevstad:** Formal analysis (supporting); validation (supporting); writing – original draft (supporting); writing – review and editing (supporting). **Michael B. Wunder:** Formal analysis (supporting); methodology (supporting); validation (supporting). **Walter Wehtje:** Conceptualization (supporting); data curation (supporting); funding acquisition (supporting); investigation (supporting); resources (equal); writing – original draft (supporting); writing – review and editing (supporting). **Lauren E. Walker:** Data curation (supporting); methodology (supporting); resources (supporting); writing – review and editing (supporting). **Douglas W. Smith:** Conceptualization (supporting); data curation (supporting); funding acquisition (supporting); project administration (supporting); resources (supporting); writing – review and editing (supporting).

## CONFLICT OF INTEREST STATEMENT

The authors declare no conflicts of interest.

## Supporting information


Appendices S1–S2

## Data Availability

We have uploaded the code for unmarked analysis to a Figshare repository here: https://figshare.com/articles/software/Scripts_for_CLNU_HDS_analysis/24002232 (McLaren, 2023). We prefer not to upload our data at this time, because the study is in progress (on year 5) and is being used for additional articles. We will make all data available at the completion of the study. It can also be made available upon request.
