# Supplementary material for: Clark's nutcracker forest community visitation: Whitebark pine maintains a keystone seed disperser
Source: Ecol Evol. 2023 Dec 21;13(12):e10813. doi: 10.1002/ece3.10813 (PMC10739129; doi:10.1002/ece3.10813)
Supplement: Supplementary file 1 — Appendices S1–S2 [file ECE3-13-e10813-s001.docx]

**Clark’s nutcracker forest community visitation:**

**Whitebark pine maintains a keystone seed disperser**

Thomas H. McLaren, Diana F. Tomback, Nels Grevstad, Michael B. Wunder

Walter Wehtje, Lauren E. Walker, and Douglas W. Smith

**Appendix S1: Supporting figures**


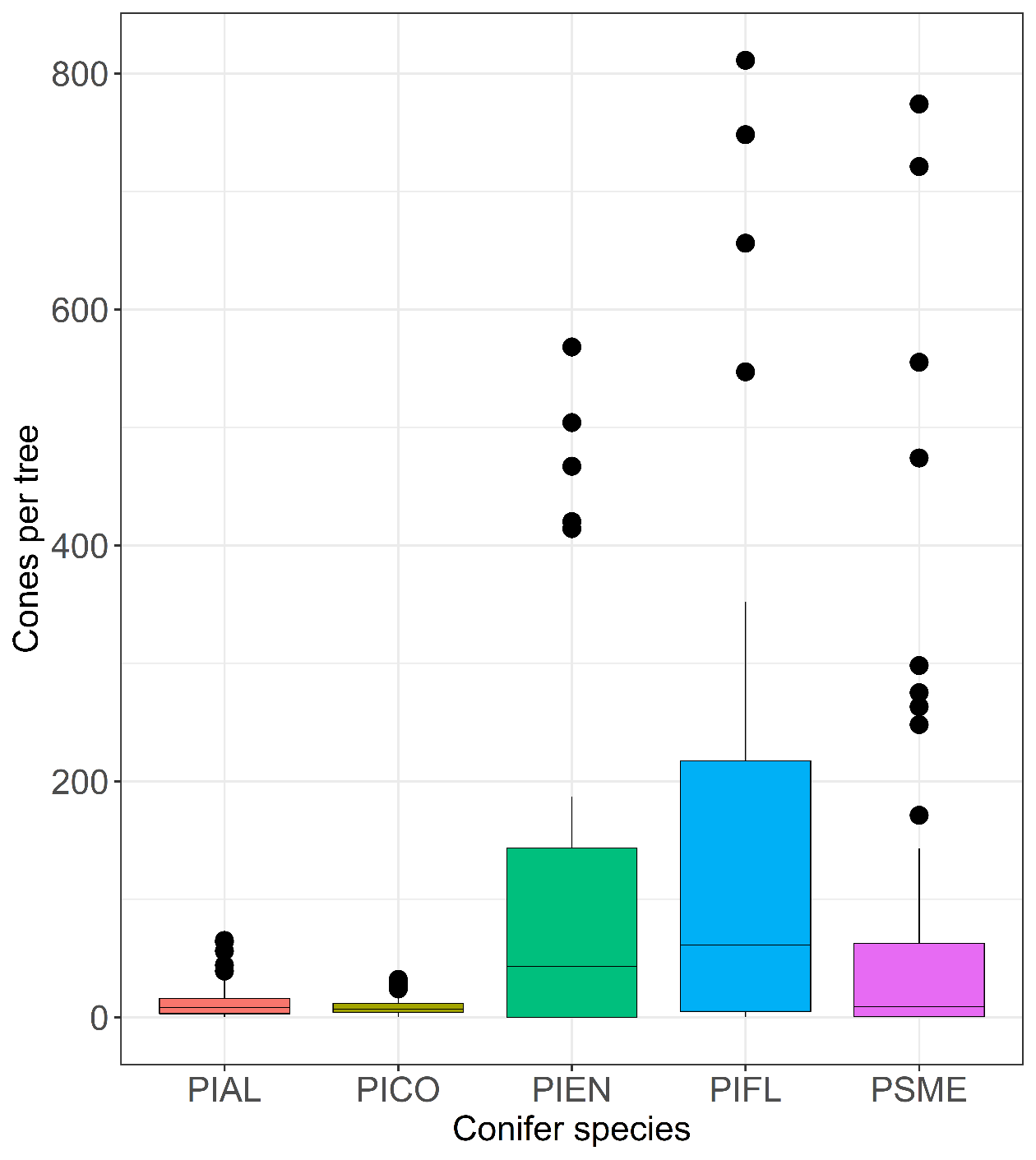


**FIGURE S1.** The total cones counted for each tree are represented as a box plot. Data from 2019 to 2021 are aggregated to show overall variability of cones per tree for each conifer species. Box plots display the first, second and third quartiles of the data for each conifer species. Whiskers indicate the largest observation that is within 1.5 times the interquartile range of the third quartile. PIAL = whitebark pine, PICO = lodgepole pine, PIEN = Engelmann spruce, PIFL = limber pine, PSME = Douglas-fir.

**
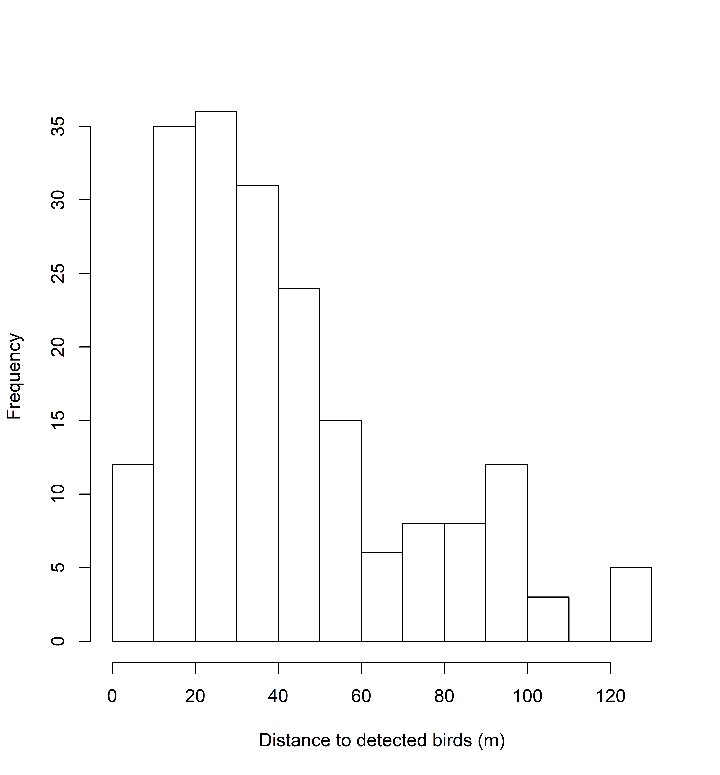
**

**Figure S2.** Histogram of distances to nutcracker detections for all Clark’s nutcracker surveys combined from 2019 to 2021. We truncated detections at a distance of 125 m from the observer to reduce the impact of more distant detections on the detection function.

**
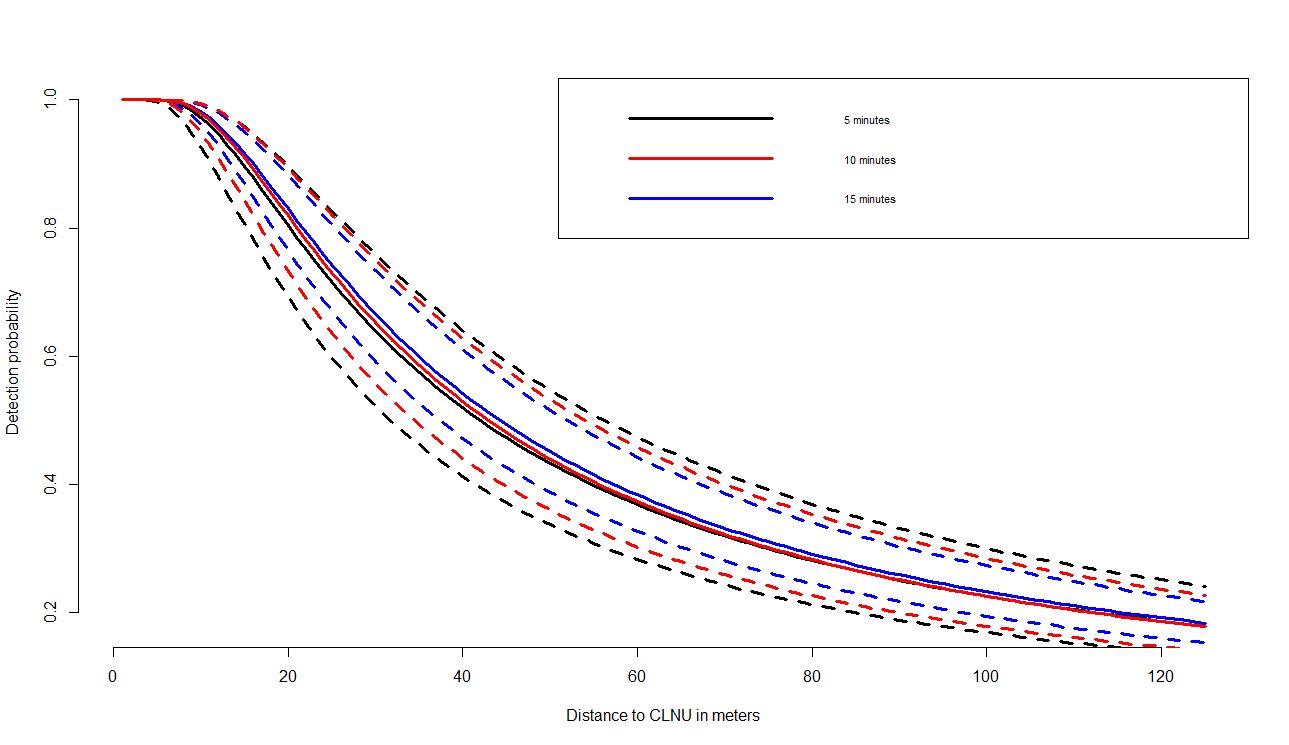
**

**Figure S3.** Given nutcrackers’ high mobility, distance sampling data could be biased due to bird movements during surveys. One way to reduce movement bias is to reduce the time length of the survey. We investigated whether shorter point count time-lengths would reduce bias by comparing the detection functions from 5, 10, and the full 15-minute point counts. We found no discernable differences among the three point count time lengths and concluded that using a shorter point count time length would not reduce any movement bias that might be present.


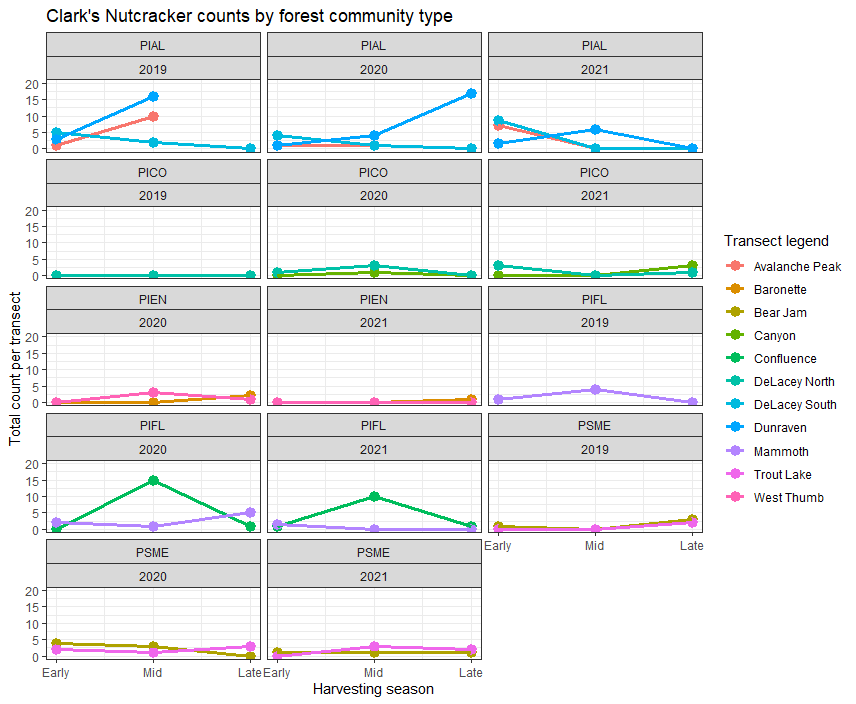


**FIGURE S4**. Counts of visually detected Clark’s nutcrackers per survey. For display purposes, counts are averaged between the two visits to each site during early harvesting season. PIAL = whitebark pine, PICO = lodgepole pine, PIEN = Engelmann spruce, PIFL = limber pine, and PSME = Douglas-fir.

**
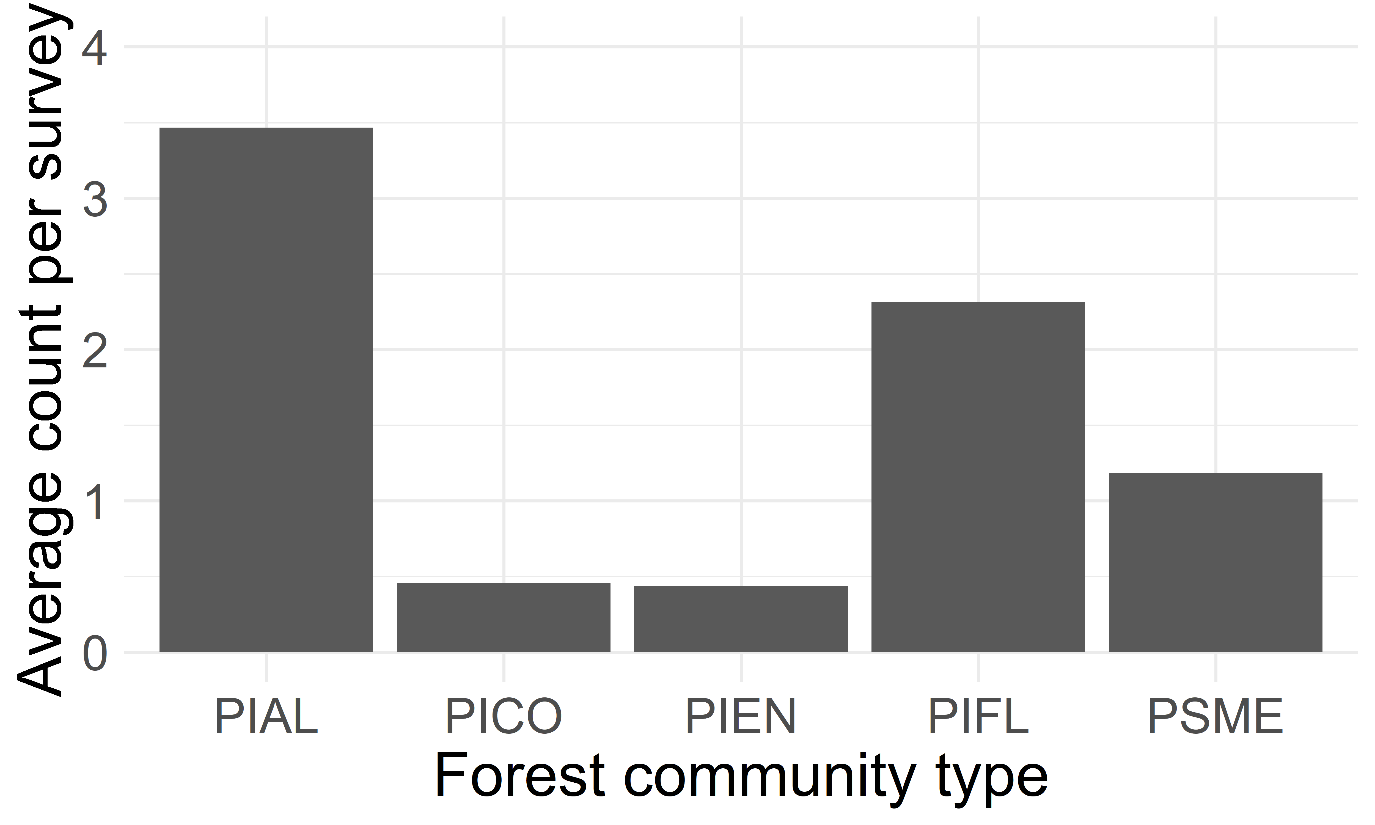
**

**Figure S5.** Summary statistics for Clark’s nutcracker survey abundance indicating the average number of nutcracker observations per survey for each forest community type for 2019-2021 field seasons. PIAL = whitebark pine, PICO = lodgepole pine, PIEN = Engelmann spruce, PIFL = limber pine, and PSME = Douglas-fir.

**Clark’s nutcracker forest community visitation:**

**Whitebark pine maintains a keystone seed disperser**

Thomas H. McLaren, Diana F. Tomback, Nels Grevstad, Michael B. Wunder

Walter Wehtje, Lauren E. Walker, and Douglas W. Smith

**Appendix S2: Supporting tables**

**Table S1.** Average number of seed harvesting and caching observations per survey, 2019-2021. During the early seed harvesting period (July through mid-August), the mid-harvesting period (early to mid-September), and the late harvesting period (early through mid-October). Values are separated by survey period and conifer species.

| Behavior | Forest community type | Early harvesting period | Mid-harvesting period | Late harvesting period |
| --- | --- | --- | --- | --- |
| Harvesting | Whitebark pine | 0.73 | 1.56 | 0.17 |
| - | Lodgepole pine | 0.1 | 0 | 0.5 |
| - | Engelmann spruce | 0 | 0 | 0 |
| - | Limber pine | 0.11 | 2.6 | 0 |
| - | Douglas-fir | 0 | 0.17 | 0.17 |
| Caching | Whitebark pine | 0 | 0 | 0.5 |
| - | Lodgepole pine | 0 | 0 | 0 |
| - | Engelmann spruce | 0 | 0.25 | 0 |
| - | Limber pine | 0 | 0.8 | 0.2 |
| - | Douglas-fir | 0 | 0.17 | 0 |

**TABLE S2** Parametric bootstrapping was used to assess model goodness of fit for the ΔAICc = 0 model of Clark’s nutcracker habitat use. We applied the ‘fitstats’ function presented in Kery and Royle (2015, Chapter 8), which leverages the parametric bootstrap function ‘parboot’ in the ‘unmarked’ package to generate three goodness of fit metrics. These metrics include the Sum of Squared Errors, Chi-square and Freeman-Tukey goodness of fit statistics, based on 500 simulated bootstrap samples. We found that two of the three bootstrapped statistics did not indicate inadequate model fit, signified by a Pr (tB > t0) above 0.05. However, in cases when bins contain few values, like in the tails of the distribution, the Chi-square statistic is known to often be biased and may not be a reliable indicator of model fit. We interpret this to support moderately good model fit. t0 = Original statistic computed from data. tB = Bootstrap sample values.

**Parametric Bootstrap Statistics**

|  | **t0** | **Mean (t0 – tB)** | **Std (t0 – tB)** | **Pr (tB > t0)** |
| --- | --- | --- | --- | --- |
| **SSE** | 354 | 48.5 | 98.1 | 0.23952 |
| **Chi-squared** | 1932 | 590.2 | 548.7 | 0.00599 |
| **Freeman Tukey** | 220 | 22.4 | 20.7 | 0.12974 |

**tB Quantiles**

|  | **0%** | **2.5%** | **25%** | **50%** | **75%** | **97.5%** | **100%** |
| --- | --- | --- | --- | --- | --- | --- | --- |
| **SSE** | 126 | 175 | 238 | 283 | 351 | 559 | 924 |
| **Chi-squared** | 943 | 1032 | 1185 | 1295 | 1410 | 1769 | 12783 |
| **Freeman Tukey** | 140 | 155 | 185 | 198 | 210 | 243 | 267 |

**Table S3.**  Numbers of stems that were breast height or taller per 500 m^2^.  The reported number for each study site was obtained by averaging the stem density values from community assessments of two 500 m^2^ (10 m x 50 m) belt transects at each study site. See text for sampling methods.

| Forest community type | Study site name | DBH Stem number |
| --- | --- | --- |
| Whitebark pine (PIAL) | Avalanche Peak | 61.0 |
|  | DeLacy South | 64.5 |
|  | Dunraven | 50.5 |
| Lodgepole pine (PICO) | Canyon | 59.5 |
|  | DeLacy North | 72.0 |
| Engelmann spruce (PIEN) | Baronette | 50.5 |
|  | West Thumb | 53.0 |
| Limber pine (PIFL) | Confluence | 2.0 |
|  | Mammoth | 9.5 |
| Douglas-fir (PSME) | Bear Jam | 11.0 |
|  | Trout Lake | 6.0 |

**Table S4** Results from monthly road survey point counts for Clark’s nutcrackers along the northern road corridor in Yellowstone National Park, conducted from November through March in 2019-2020 and 2020-2021. Surveys occurred monthly with the exception of November 2019, when two surveys were completed, and December 2020, when no surveys were conducted. Forest community types were identified using Yellowstone GIS habitat layers, which were ground-truthed as point count stations were established. Nutcrackers were detected at 70% of the points in the winter of 2019 and 100% of points in 2020. ‘A’ indicates sites with nutcracker detections in 2019-2020; ‘B’ indicates sites with nutcracker detections in 2020-2021.

| **Survey Point** | **Forest community type** | **Nov.**  (1^st^ survey) | **Nov.**  (2^nd^ survey)  (Not conducted in 2020-2021) | **Dec.**  (Not conducted in 2019-2020) | **Jan.** | **Feb.**  (Not conducted in 2020-2021) | **Mar.** |
| --- | --- | --- | --- | --- | --- | --- | --- |
| 1 | Limber pine/ Douglas-fir |  |  |  | B |  | B |
| 2 | Limber pine/ Douglas-fir | B | A | B |  |  |  |
| 3 | Douglas-fir | B | A |  | A |  | A, B |
| 4 | Douglas-fir |  |  |  | B |  | A |
| 5 | Limber pine/ Douglas-fir |  |  |  | B | A | A |
| 6 | Lodgepole pine |  |  | B | B |  | B |
| 7 | Subalpine fir | B |  | B |  |  | B |
| 8 | Engelmann spruce | B |  | B | B |  | A, B |
| 9 | Subalpine fir | B |  |  | B | A |  |
| 10 | Engelmann spruce/ Subalpine fir |  |  |  |  | A | A, B |

**References used in Appendix S2**

Kéry, M., and J. A. Royle. 2015. *Applied Hierarchical Modeling in Ecology: Analysis of Distribution, Abundance and Species Richness in R and BUGS:Volume 1.* Academic press. <https://doi.org/10.1016/C2015-0-04070-9>
